# Comparative performance analysis of binary variants of FOX optimization algorithm with half-quadratic ensemble ranking method for thyroid cancer detection

**DOI:** 10.1038/s41598-023-46865-8

**Published:** 2023-11-10

**Authors:** Rohit Sharma, Gautam Kumar Mahanti, Ganapati Panda, Adyasha Rath, Sujata Dash, Saurav Mallik, Zhongming Zhao

**Affiliations:** 1 Department of Electronics and Communication Engineering, NIT, Durgapur, 713209 India; 2 Department of Electronics and Communication Engineering, C.V. Raman Global University, Bhubaneswar, 752054 India; 3 Department of Computer Science and Engineering, C.V. Raman Global University, Bhubaneswar, 752054 India; 4 Department of Information Technology, Nagaland University, Dimapur, India; 5 Department of Environmental Health, Harvard T. H. Chan School of Public Health, Boston, MA USA; 6 Center for Precision Health, School of Biomedical Informatics, The University of Texas Health Science Center at Houston, Houston, TX 77030 USA

**Keywords:** Computational biology and bioinformatics, Engineering, Mathematics and computing

## Abstract

Thyroid cancer is a life-threatening condition that arises from the cells of the thyroid gland located in the neck’s frontal region just below the adam’s apple. While it is not as prevalent as other types of cancer, it ranks prominently among the commonly observed cancers affecting the endocrine system. Machine learning has emerged as a valuable medical diagnostics tool specifically for detecting thyroid abnormalities. Feature selection is of vital importance in the field of machine learning as it serves to decrease the data dimensionality and concentrate on the most pertinent features. This process improves model performance, reduces training time, and enhances interpretability. This study examined binary variants of FOX-optimization algorithms for feature selection. The study employed eight transfer functions (S and V shape) to convert the FOX-optimization algorithms into their binary versions. The vision transformer-based pre-trained models (DeiT and Swin Transformer) are used for feature extraction. The extracted features are transformed using locally linear embedding, and binary FOX-optimization algorithms are applied for feature selection in conjunction with the Naïve Bayes classifier. The study utilized two datasets (ultrasound and histopathological) related to thyroid cancer images. The benchmarking is performed using the half-quadratic theory-based ensemble ranking technique. Two TOPSIS-based methods (H-TOPSIS and A-TOPSIS) are employed for initial model ranking, followed by an ensemble technique for final ranking. The problem is treated as multi-objective optimization task with accuracy, F2-score, AUC-ROC and feature space size as optimization goals. The binary FOX-optimization algorithm based on the $V_1$ transfer function achieved superior performance compared to other variants using both datasets as well as feature extraction techniques. The proposed framework comprised a Swin transformer to extract features, a Fox optimization algorithm with a V1 transfer function for feature selection, and a Naïve Bayes classifier and obtained the best performance for both datasets. The best model achieved an accuracy of 94.75%, an AUC-ROC value of 0.9848, an F2-Score of 0.9365, an inference time of 0.0353 seconds, and selected 5 features for the ultrasound dataset. For the histopathological dataset, the diagnosis model achieved an overall accuracy of 89.71%, an AUC-ROC score of 0.9329, an F2-Score of 0.8760, an inference time of 0.05141 seconds, and selected 12 features. The proposed model achieved results comparable to existing research with small features space.

## Introduction

Thyroid cancer typically begins as a small lump or nodule in the thyroid gland and can eventually spread to nearby lymph nodes or other areas of the body. Common symptoms include a palpable neck lump, swallowing difficulties, voice changes, and enlarged lymph nodes. The prognosis for thyroid cancer is generally positive, especially for early-stage and well-differentiated cases, with high survival rates^1,2^. The machine learning (ML) tools have the potential to facilitate the early detection of thyroid cancer. Utilizing ML tools can support healthcare professionals in making accurate diagnoses, resulting in improved patient outcomes and potentially reducing the need for invasive procedures. By incorporating machine learning into the diagnostic process, thyroid cancer can be detected more quickly and effectively, allowing for earlier treatment and better patient outcomes^3–5^.

## Literature review

Recent studies have shown the promising performance of ultrasound images-based computer-aided diagnosis (CAD) tools for detecting thyroid cancer^5–11^. Chi et al.^8^ proposed pre-trained GoogleNet for feature extraction and achieved 79.36% accuracy for thyroid nodule detection. Similarly, Sai and others^9^ utilized pre-trained GoogleNet and VGG16 for thyroid cancer detection, achieving 79.36% and 77.57% accuracy, respectively. In their research, Nguyen et al.^10,11^ employed spatial and frequency domain analysis to detect cancerous nodules in the thyroid region. The authors used convolution neural network (CNN) and fast fourier transform (FFT) for knowledge extraction. They employed a weighted cross-entropy function and voting ensemble learning for class imbalance. The proposed methods provided 90.88% and 92.05% accuracies. Sharma et al.^5^ used an ensemble learning model for thyroid cancer diagnosis using ultrasound images. The authors used the hunger games search (HGS) to get ensemble weightes for mixers and transformer models. Researchers proposed distance correlation for weighting the criteria whereas TOPSIS (Technique for Order of Preference by Similarity to Ideal Solution) is used for benchmarking the models. The researchers achieved 82.18% accuracy on thyroid ultrasound dataset. Similar to ultrasound images, histopathological images are successfully analysed for thyroid cancer detection using ML methods^12–16^. Wang et al.^12^ have evaluated the performance of VGG-19 and Inception-ResNet-v2 to classify thyroid nodule in histological images. VGG-19 achieved the best average diagnostic accuracy of 97.34%, whereas Inception-ResNet-v2 obtained 94.42% accuracy. Using deep neural networks and transfer learning methods, the models suggested by Buddhavarapu et al.^13^ introduced an automated classification model for thyroid histopathology images. Popular pre-trained CNN architectures, VGGNet, ResNet, InceptionNet, and DenseNet, are used in transfer learning for fine-tuning and extracting the important features. The results have shown the applications of transfer learning for histopathology image analysis for thyroid case study, with DenseNet achieving the best accuracy of 100% using the 80:20 data split strategy on a private dataset. A CAD system proposed by Jothi et al.^14^ for segmenting and classifying H &E stained thyroid histopathology images into normal thyroid or PTC thyroid. The model employed particle swarm optimization based on Otsu’s multilevel thresholding to segment the images and manually choose a binary image comprising the nuclei. The researchers suggested a novel closest-matching-rule (CMR) method to categorize new test samples. The proposed model achieved an accuracy of 99.54% on a private dataset. Bohland et al.^15^ proposed feature extraction and deep learning models for the categorizing histopathological thyroid images into papillary thyroid carcinoma (PTC) and non-papillary thyroid carcinoma (NPTC). The proposed methods provided diagnostic accuracy of 89.10% and 89.70% for deep learning and feature extraction techniques, respectively. Do et al.^16^ trained and evaluated the Inception-v3 model on Tharun Thompson and Nikiforov datasets and achieved accuracies of 85.73% and 72.65 %, respectively.

The curse of dimensionality presents significant obstacles for machine learning models when dealing with high-dimensional data. Overcoming the curse of dimensionality is vital for machine learning models to effectively handle high-dimensional data^17,18^. Meta-heuristic algorithms are well-suited for a wide range of optimization tasks due to their ability to balance exploitation and exploration^19^. Binary meta-heuristic algorithms are widely used for feature selection in machine learning. These algorithms are designed to solve optimization problems to select an optimal feature subset that maximizes the model’s performance^20,21^. Transfer functions are essential to convert continuous values into binary representations in binary optimization. They enable binary optimization algorithms to operate effectively on binary strings and perform operations like crossover and mutation. Common transfer functions used in binary optimization include the sigmoid, threshold, step, and linear functions^22^. The sigmoid function maps continuous values to binary values between 0 and 1, while the threshold function assigns 1 to values above a fixed threshold and 0 to values below it. The step function uses a threshold to convert values above it to 1 and values below it to 0; the linear function scales and maps values using a linear transformation. The choice of transfer function depends on the specific requirements and data characteristics of the binary optimization problem, as different functions can impact the exploration and exploitation abilities of the algorithm. Selecting or designing a transfer function that aligns with the problem’s objectives and constraints is essential^23,24^. Various statistical tests can be used to compare the performance of meta-heuristic algorithms. The wilcoxon rank-sum test and Friedman test are non-parametric tests suitable for comparing two algorithms on multiple instances and multiple algorithms on various instances, respectively. The Student’s t-test is a parametric test that compares the means of two algorithms on a single instance if the data follows a normal distribution. The ANOVA is useful for comparing the means of multiple algorithms on a single instance. The choice of test depends on the data’s nature, the number of algorithms, and the research question while considering the assumptions of each test and their compatibility with the data^25^.

Instead of relying solely on statistical tests, multi-criteria decision making (MCDM) techniques offer an alternative approach to rank meta-heuristic algorithms. These techniques take into account multiple criteria or performance measures to establish rankings that consider various aspects of algorithm performance^26,27^. Krohling et al.^26^ proposed A-TOPSIS technique for evolutionary algorithm ranking, whereas in Ref.^27^ Hellinger TOPSIS (H-TOPSIS) and TODIM approaches are used for the ranking purpose. Since MCDM methods employ different mechanisms, it can be challenging to provide conclusive evidence for the ranking produced by a particular MCDM method compared to others. Consequently, these techniques may yield different ranks for the same application, adding complexity to determining the most reliable ranking. To address the issue of varying rankings produced by different MCDM methods, a proposed approach is to use a compromised ensemble MCDM method based on half-quadratic theory^28^. This method aims to establish confidence or consensus among the different MCDM rankings. Combining the rankings through a ensemble approach seeks to achieve a more robust and reliable overall ranking that considers the strengths and weaknesses of each MCDM method. The novel contribution of the proposed research is as follows:-

1. Transformer-based pre-trained models (specifically DeiT and Swin Transformer) extract features from thyroid ultrasound and histopathology images. These models are employed to capture essential patterns and information in the images, enabling the extraction of relevant features that can be used for further analysis or classification task.
2. The extracted features are transformed into lower dimensional space using the locally linear embedding technique (LLE).
3. Eight S and V transfer function based binary FOX optimization techniques are analyzed for the selection og features along with Naive Bayes as a classifier. The classifier is evaluated for 5-fold cross-validation with a stratified oversampling technique in order to balance the datasets. The optimization technique is evaluated as a weighted average multi-objective optimization method. The optimization goals are Accuracy, F2-score, AUC-ROC Score and selected feature space size.
4. A-TOPSIS and H-TOPSIS are used to evaluate the ranking of the models evaluated on test datasets in the initial step.
5. Two ranking techniques are further ensembled using the half-quadratic distance and final ranks are evaluated.
6. The best-ranked transfer function-based FOX algorithm is used for feature selection with more epochs to improve the performance metrics, and the obtained model is evaluated and compared with the existing research.

## Material and methodology

### Datasets

The proposed framework undergoes evaluation using two distinct imaging datasets. These two datasets are obtained using ultrasound and histopathological modalities. The ultrasound dataset used in the study is sourced from the Thyroid Digital Image Database (TDID), which is publicly accessible^29^. It includes 347 samples from 298 patients, with ultrasound images of dimensions 560X360 pixels. Each thyroid ultrasound image includes nodule localization details and TIRAD scores. TIRAD scores range from 1 to 5, indicating the condition of the thyroid nodule, with scores less than 4 representing benign nodules and scores greater than 4 indicating malignant nodules. The Tharun Thompson histopathology dataset comprises 156 thyroid tumors provided by two medical centers^30^. Whole slide images of the tumors are scanned and converted into 8-bit color images. Two expert pathologists categorized each image independently, reaching a predictive consensus for each case. The dataset tumors are classified into two distinct groups using five entities: non-papillary thyroid carcinoma-like (NPTC-like) and papillary thyroid carcinoma (PTC-like). The PTC-like category encompasses two types of thyroid nodule abnormalities: follicular thyroid adenoma (FA) and follicular thyroid carcinoma (FTC). Meanwhile, the NPTC-like group includes follicular variant papillary thyroid carcinoma (FVPTC), classical papillary thyroid carcinoma (PTC) and noninvasive follicular thyroid neoplasm with papillary-like nuclear features (NIFTP). Out of the 156 tumors, 147 had ten images extracted from neoplastic areas, while the remaining nine cases had fewer images due to the smaller size of the neoplasm areas. There are a total of 1700 histopathological images after over-sampling in this dataset.

### Methodology

**Figure 1:**
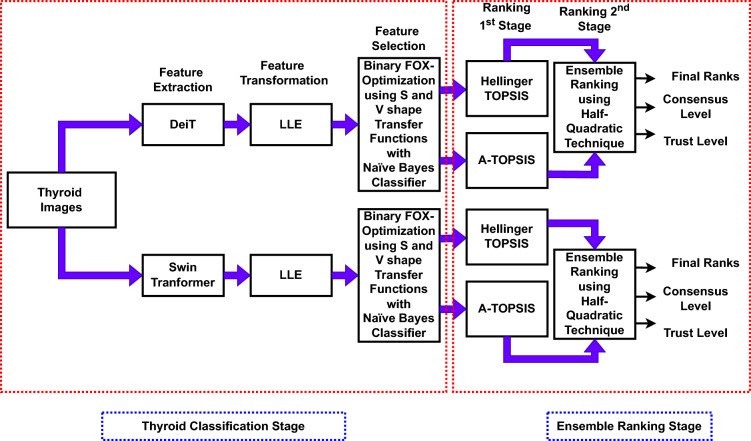
A framework for evaluating and ranking the binary variants of FOX optimization algorithms for feature selection on thyroid datasets using transformers and ensemble MCDM technique.

The research work depicted in Fig. 1 encompasses the overall process. Initially, two pre-trained models based on deep transformer learning (DeiT and Swin Transformer)^31,32^ are employed for feature extraction. The proposed study used the DeiT-Small model pre-trained using ImageNet-1K dataset containing 22 million parameters. A pre-trained Swin Transformer model, namely the Swin-S version, is employed in this case. This model comprises a linear projection dimension of 96 and 50 million tunable parameters.

The extracted feature vector is immediately used in the subsequent machine learning process in ultrasound image case study. However, 45 patch images are extracted from a single histopathological image for the second dataset, and feature vectors are extracted from every patch. These feature vectors are combined through fusion and provided to the LLE block for further dimensionality reduction. The LLE^18^ is a nonlinear approach to decrease the dimensionality of data. Its objective is to capture the local connections between data points and their neighbouring points, which are then employed to create a lower-dimensional data representation.

#### Binary FOX-optimization algorithms

After feature transformation, feature selection is performed to reduce the dimesnionality of the feature space further. The FOX-optimization technique is employed for the selection of important features.

Eight different binary variants of the FOX optimization algorithm are proposed and utilized in order to optimize the cost function value for feature selection. The cost function value is calculated using the Eq. (1).

1 $$\begin{aligned} CF = -(a\times Acc+b\times F_{1}+c\times AS-d\times (CSF/FSD). \end{aligned}$$

Here *CF* is the cost function to be evaluated with constraint given in Eq. (2). Here *a*, *b*, *c*, and *d* are the weighting hyper-parameters for the individual cost functions.

2 $$\begin{aligned} a+b+c+d = 1. \end{aligned}$$

The task is considered as a minimization problem. The algorithms are executed 30 times, and the mean and standard deviation values are determined for accuracy, F2-Score, AUC-ROC score, inference time, feature selected, cost function value, and elapsed time at each iteration. The *CSF* refers to the cardinality or number of selected features, while *FSD* represents the dimension of the feature space.

**Figure 2:**
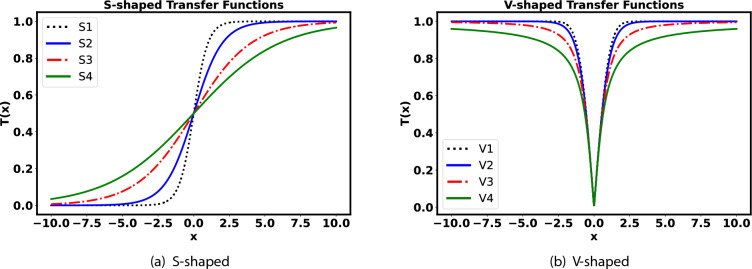
Transfer Functions.

- *FOX-Optimization*: The hunting behavior of foxes inspires the FOX optimization algorithm^33^. It includes methods for assessing the distance between the fox and its target, enabling efficient leaps or jumps in the optimization process. The algorithm calculates a new position for the fox based on factors such as the jump value, direction range and distance to the prey. The algorithm mimics the red fox’s strategy of randomly searching for prey in snowy conditions by relying on its ability to hear the ultrasounds emitted by the prey. The fox estimates the distance to the prey by analyzing the sound and calculates the precise jump needed to capture it. The FOX algorithm initializes a population of search agents represented by a matrix and calculates their fitness using benchmark functions. It balances exploration and exploitation phases using a random variable, and the best fitness and position values are determined throughout the iterations. The algorithm gradually decreases the search performance based on the best position, enabling the effective exploration and activation of different phases.
- *Binary Variants using S and V Shape Transfer Functions*: The search process results in new positions for the red fox are in continuous form. However, these continuous positions need to be converted into binary values. This transformation is achieved by applying S-shaped and V-shaped transfer functions to each dimension of the positions^34^. These transfer functions guide the red fox to move with binary locations. Four sigmoidal (S-shaped) transfer functions are used to convert the real values of the fox position into probability values ranging from 0 to 1. The transfer function values are calculated using Eqs. (3), (4), (5) and (6).
  3 $$\begin{aligned} S_{1}\rightarrow T(X_{i}^{k}(u))= & {} 1/(1+e^{-2X_{i}^{k}(u)}) \end{aligned}$$
  4 $$\begin{aligned} S_{2}\rightarrow T(X_{i}^{k}(u))= & {} 1/(1+e^{-X_{i}^{k}(u)}) \end{aligned}$$
  5 $$\begin{aligned} S_{3}\rightarrow T(X_{i}^{k}(u))= & {} 1/(1+e^{(-X_{i}^{k}(u)/2)}) \end{aligned}$$
  6 $$\begin{aligned} S_{4}\rightarrow T(X_{i}^{k}(u))= & {} 1/(1+e^{(-X_{i}^{k}(u)/3)}) \end{aligned}$$
  Here, $X_{i}^{k}$ indicates the position of red-fox *i* with $u^{th}$ iteration in $k^{th}$ dimension. The continuous values are converted into binary versions based on the condition in Eq. (7). The *rand* is a random number lies between 0 and 1.
  7 $$\begin{aligned} X^{k}_{i}(u+1) = \left\{ \begin{array}{cl} 1 &{}: \ rand \ge T(X^{k}_{i}(u)) \\ 0 &{}: \ rand < T(X^{k}_{i}(u)) \end{array} \right. \end{aligned}$$
  Simillarly four V shaped transfer functions are given in Eqs. (8), (9), (10) and (11).
  8 $$\begin{aligned} V_{1}\rightarrow T(X_{i}^{k}(u))= & {} \left| erf(\sqrt{\pi }/2X_{i}^{k}(u)) \right| \end{aligned}$$
  9 $$\begin{aligned} V_{2}\rightarrow T(X_{i}^{k}(u))= & {} \left| \tanh (X_{i}^{k}(u)) \right| \end{aligned}$$
  10 $$\begin{aligned} V_{3}\rightarrow T(X_{i}^{k}(u))= & {} \left| X_{i}^{k}(u)/\sqrt{1+ X_{i}^{k}(u)} \right| \end{aligned}$$
  11 $$\begin{aligned} V_{4}\rightarrow T(X_{i}^{k}(u))= & {} \left| 2(\arctan (\pi X_{i}^{k}(u)/2))/\pi \right| \end{aligned}$$
  The V-shaped transfer function’s threshold directions are represented mathematically in Eq. (12). If the *rand* value is less than the transfer function value, then each binary value in the position vector is inversed; otherwise, there is no change in the fox position from the last iteration.
  12 $$\begin{aligned} X^{k}_{i}(u+1) = \left\{ \begin{array}{cl} X^{k}_{i} &{}: \ rand \ge T(X^{k}_{i}(u)) \\ (X^{k}_{i})^{-1} &{}: \ rand < T(X^{k}_{i}(u)) \end{array} \right. \end{aligned}$$
  The S and V shaped Transfer functions used in this research are shown in Fig. 2.

### Model ranking

#### Half quadratic based ensemble ranking technique

Comparing algorithms in evolutionary computation poses a significant challenge. Typically, algorithms are executed multiple times on various benchmark problems, and the results are subsequently analyzed using statistical hypothesis tests. These tests aim to identify any performance differences among the algorithms. However, a crucial issue arises: if differences exist, how can we determine which algorithm is the best? To address this, pairwise comparisons between the algorithms need to be conducted, which can be a laborious task, especially when dealing with a large number of algorithms. Not only is it time-consuming to compare each pair of algorithms, but there is also an increased risk of making errors in the process^26^. Instead of statistical tests, MCDM techniques can be used for ranking the evolutionary algorithms^27^. A significant point of contention in this field is that different MCDM methods generate distinct and potentially contradictory rankings even when applied to the same input. Consequently, it becomes crucial to determine an overall aggregated ranking of alternatives in order to reconcile these differences.

The ensemble method put forward in this study applies to any number of MCDM methods^28^.For example, consider a scenario where *N* different MCDM techniques are used to benchmark the a set of *L* alternatives based on *p* criteria. A simple way to obtain the aggregated ranks ($S^*$) is to reduce its Euclidean seperation from every calculated ranking. The task of minimization can be performed as follows:

13 $$\begin{aligned} min_{S^{*}}\frac{1}{2}\sum _{n-1}^{N}h(\left\| S^{n}-S^{*}\right\| _{2}) \end{aligned}$$

In the given equation, *N* represents the total number of MCDM methods, while $S^n$ refers to the ranking generated by the $n^{th}$ MCDM method. The h(.) is a half-quadratic (HQ) function. The half-quadratic programming is used to solve this non-convex problem.

#### Consensus index

The consensus index provides a measure of agreement among the ranking methods employed, enabling the calculation of the similarity between the aggregated ranking and every individual ranking. The Consensus Index (*C*) value is calculated using Eq. (14).

14 $$\begin{aligned} C(S^{*})= & {} \frac{1}{KN}\sum _{k=1}^{K}\sum _{n=1}^{N}q_{kn} \end{aligned}$$

15 $$\begin{aligned} q_{kn}= & {} \frac{\mathcal {N}_{\sigma }(S_{k}^{*}-S_{k}^{n})}{\mathcal {N}_{\sigma }(0)} \end{aligned}$$

$\mathcal {N}_{\sigma }(.)$ $\rightarrow$ probability density function of Gaussian distribution with zero mean and standard deviation $\sigma$. The $\mathcal {N}_{\sigma }(0)$ used for normalization and hence the value of $q_{kn}$ lies in [0, 1].

#### Trust level

The trust level serves as a measure of the credibility of the ensemble ranking. If a particular MCDM ranking differs from the most of rankings significantly, it is assigned with a smaller weight value, resulting in a reduced impact on the final ranking. This lower-weighted method diminished the influence on the trust level. Considering these factors, the trust level can be calculated as a reflection of the weighting assigned to each method. The trust level is calculated using Eq. (16).

16 $$\begin{aligned} T(S^{*}) = \frac{1}{K}\sum _{k=1}^{K}\sum _{n=1}^{N}w_{n}q_{kn} \end{aligned}$$

where

$$\begin{aligned} w_n = \frac{\alpha _n }{\sum _{j=1}^{N}\alpha _{j}} \end{aligned}$$

*n* $\rightarrow$ 1,2,3,....,*N* and $\alpha$ $\epsilon$ $S^n$, where n is the ranking MCDM technique number involved in ensemble process and $\alpha$ is the half-quadratic auxiliary variable.

Two MCDM techniques, A-TOPSIS and H-TOPSIS, are employed in the initial stage of rank evaluation. The ranks obtained from these two techniques are subsequently utilized in the final rank calculation process using the half-quadratic ensemble method. These methods processed a decision matrix that contains rankings obtained based on average values and standard deviations values. The purpose is to provide a method that assists in ranking the best algorithms when applied to different alterantives that are evaluated using the average and standard deviations values as criteria.

#### A-TOPSIS

**Figure 3:**
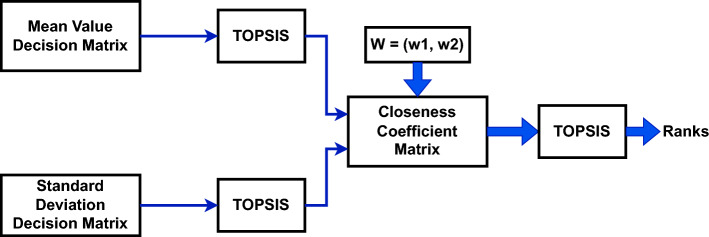
A-TOPSIS method.

The A-TOPSIS method is illustrated in Fig. 3. The steps for A-TOPSIS ranking are as follow:

1. Normalize the mean and standard deviation decision matrices. The criteria and alternatives are arranged in columns and rows, respectively.
2. Determine the positive ideal solutions and negative ideal solutions for each matrix by identifying the optimal and suboptimal values for the given criteria.
3. Compute the Euclidean distances between each alternative and both the positive ideal solution and the negative ideal solution.
4. Obtain the relative closeness of every alternative in relation to the positive ideal and negative ideal solution.
5. Once the relative-closeness coefficient vectors are computed for these two decision matrices, the final decision matrix is obtained by combining the two vectors of relative-closeness coefficients. The weights are assigned to both the vectors given as:
  $$\begin{aligned} W = (w_1, w_2) = (w_{mean}, w_{standard \,deviation}) \end{aligned}$$
  These weights are used to compute the weighted normalized decision matrix.
6. Perform the same steps from 1 to 5 on the weighted normalized decision matrix and obtain the global relative-closeness coefficient
7. Arrange the alternatives based on the values of relative closeness coefficient. The top-ranked alternatives are those with largest values.

#### H-TOPSIS

1. Generate a decision matrix using the average values derived from the evolutionary runs.
2. Calculate the positive ideal solution (*PIS*) and negative ideal solution (*NIS*) considering problem of minimization as follow: *PIS* $\rightarrow$ $g_j^+$ $\leftarrow$ $g_{ij}$ $=$ $min_i(\mu _{ij})$ for $j = 1,2,...,K$ and $i = 1,2,3,...,N$ *NIS* $\rightarrow$ $g_j^-$ $\leftarrow$ $g_{ij}$ $=$ $max_i(\mu _{ij})$ for $j = 1,2,...,K$ and $i = 1,2,3,...,N$ with $N(\mu _{ij}, \sigma _{ij})$, where $\mu _{ij}$ and $\sigma _{ij}$ are mean and standard deviation values, respectively.
3. Suppose *h* represents the probability density function of a Normal distribution $\mathcal {N}(\mu _1, \sigma _1)$, and *i* represents the pdf of a normal distribution $\mathcal {N}(\mu _2, \sigma _2)$. The Hellinger distance between *h* and *i* can be expressed as follows:
  17 $$\begin{aligned} D_H(h,i) = \sqrt{1-\sqrt{\frac{2\sigma _1\sigma _2}{\sigma _1^{2}+\sigma _2^{2}}}\exp \left( \frac{-0.25(\mu _1^2+\mu _2^2)}{\sigma _1^{2}+\sigma _2^{2}} \right) } \end{aligned}$$
4. Compute the separation metrics for each alternative by calculating the distances from the positive ideal solutions $g^+$ and the negative ideal solutions $g^-$. The distances are calculated using Eqs. (18) and (19).
  18 $$\begin{aligned} dist_i^+= & {} \sum _{j = 1}^{n} w_j D_H(g^+_j, g_{ij}) \end{aligned}$$
  19 $$\begin{aligned} dist_i^-= & {} \sum _{j = 1}^{n}w_jD_H(g^-_j, g_{ij}) \end{aligned}$$
  with $i=1,2.....,N$ , where *N* is the number of alteratives.
5. For algorithm comparison, the equal weights are used, so Eqs. (18) and (19) changed into new Eqs. (20) and (21).
  20 $$\begin{aligned} dist_i^+= & {} \sum _{j = 1}^{n} D_H(g^+_j, g_{ij}) \end{aligned}$$
  21 $$\begin{aligned} dist_i^-= & {} \sum _{j = 1}^{n}D_H(g^-_j, g_{ij}) \end{aligned}$$
6. Calculate the relative closeness coefficient for each alternative concerning both the positive and negative ideal solutions with the help of Eq. (22).
  22 $$\begin{aligned} \xi _i = \frac{dist_i^-}{dist_i^++dist_i^-} \end{aligned}$$
7. Arrange the alternatives in order based on their relative closeness coefficients. The top-ranked alternatives with higher values are considered the best choices as they are nearer to the PIS.

## Simulation based experimental results

The framework is applied to two thyroid disease image datasets: ultrasound and histopathological. Both types of images are processed using the proposed approach. Feature extraction is performed using pre-trained transformer models, namely DeiT-small and Swin-Transformer-small. The feature vector sizes for ultrasound images are 384 and 1025 for the DeiT-Small and Swin-Transformer models, respectively. On the other hand, histopathological images consist of 45 patch images, resulting in feature vector sizes of 17280 and 46125 after feature fusion of features obtained from DeiT and Swim-transformer models, respectively. The LLE is applied to reduce the features, resulting in feature vectors of size 200 for ultrasound datasets and 1000 for histopathological datasets. The reduced feature set is provided to train the Naive Bayes classifier along with S and V shaped binary FOX-optimization for wrapper-based feature selection. The reduced features, obtained through LLE, are utilized in conjunction with the Naive Bayes classifier for classification tasks. Additionally, the S-shaped and V-shaped binary FOX-optimization algorithms are employed as wrapper-based feature selection techniques. These optimization algorithms aid in selecting the most relevant features from the reduced feature set, further reducing the cost value. By combining the feature selection process with the Naive Bayes classifier, the framework aims to compare the performance of the binary variant of the FOX algorithm for feature selection in a classification task. The classification problem is a multi-objective optimization task with four performance parameters (accuracy, F2-Score, AUC-ROC score, and cardinality of feature selected) to be optimized. The algorithms are applied 30 times, and the mean and standard deviation values of different performance parameters (accuracy, F2-Score, AUC-ROC score, inference time, cardinality of feature selected and elapsed time) are evaluated for both datasets. The optimization algorithms run for 100 epochs with 30 initial positions.

**Table 1:** Calculated values of cost function, Hellinger-TOPSIS ranks, A-TOPSIS ranks, $R^*$ and final ranks along with trust level and consensus index for ultrasound images.

Sr. No.	Transfer function	Cost value	H-TOPSIS ranking	A-TOPSIS ranking	R*	Final ranks
1.	S1	− 61.2401 ± 0.5131	6	7	6.5000	6
2.	S2	− 61.0373 ± 0.4052	5	5	5.0000	5
3.	S3	− 60.8880 ± 0.5404	7	6	6.5000	7
4.	S4	− 60.8589 ± 0.3989	8	8	8.0000	8
5.	V1	− 62.9983 ± 0.4413	1	1	1.0000	1
6.	V2	− 63.1388 ± 0.5027	2	2	2.0000	2
7.	V3	− 62.9797 ± 0.4063	3	3	3.0000	3
8.	V4	− 62.8899 ± 0.5982	4	4	4.0000	4
					Consensus index	0.7501
					Trust level	0.7501

The features are extracted using DeiT model and naive bayes is used as a classifer.

**Table 2:** Calculated values of cost function, Hellinger-TOPSIS ranks, A-TOPSIS ranks, $R^*$ and final ranks along with trust level and consensus index for ultrasound images.

Sr. No.	Transfer function	Cost value	H-TOPSIS ranking	A-TOPSIS ranking	R*	Final ranks
1.	S1	− 65.4736 ± 0.3480	4	3	3.5000	3
2.	S2	− 65.2897 ± 0.4157	6	6	6.0000	6
3.	S3	− 64.9741 ± 0.3789	8	8	8.0000	8
4.	S4	− 64.8440 ± 0.3567	7	7	7.0000	7
5.	V1	− 67.0822 ± 0.2613	2	1	1.5000	1
6.	V2	− 67.0001 ± 0.4257	5	5	5.0000	5
7.	V3	− 66.8351 ± 0.3790	3	4	3.5000	4
8.	V4	− 66.5852 ± 0.3124	1	2	1.5000	2
					Consensus index	0.5677
					Trust level	0.5677

The features are extracted using Swin model and naive bayes is used as a classifer.

**Table 3:** Calculated values of cost function, Hellinger-TOPSIS ranks, A-TOPSIS ranks, $R^*$ and final ranks along with trust level and consensus index for histopathology images.

Sr. No.	Transfer function	Cost value	H-TOPSIS ranking	A-TOPSIS ranking	R*	Final ranks
1.	S1	− 53.4090 ± 0.3733	5	6	5.5000	5
2.	S2	− 53.2833 ± 0.4905	6	5	8.0000	6
3.	S3	− 53.4550 ± 0.4442	8	8	7.0000	8
4.	S4	− 53.4728 ± 0.3993	7	7	1.0000	7
5.	V1	− 53.8995 ± 0.3509	1	1	3.0000	1
6.	V2	− 53.6983 ± 0.5234	4	2	2.5000	3
7.	V3	− 53.9096 ± 0.5490	2	3	3.5000	2
8.	V4	− 53.9172 ± 0.4263	3	4	5.5000	4
					Consensus index	0.6592
					Trust level	0.6592

The features are extracted using DeiT model and naive bayes is used as a classifer.

**Table 4:** Calculated values of cost function, Hellinger-TOPSIS ranks, A-TOPSIS ranks, $R^*$ and final ranks along with trust level and consensus index for histopathology images.

Sr. No.	Transfer function	Cost value	H-TOPSIS ranking	A-TOPSIS ranking	R*	Final ranks
1.	S1	− 57.0562 ± 0.3710	4	6	5.0000	6
2.	S2	− 56.9526 ± 0.4213	6	1	3.5000	4
3.	S3	− 57.0480 ± 0.6849	8	8	8.0000	8
4.	S4	− 57.0820 ± 0.3767	7	7	7.0000	7
5.	V1	− 56.7903 ± 0.3324	2	3	2.5000	1
6.	V2	− 56.7581 ± 0.5701	5	4	4.5000	5
7.	V3	− 56.7129 ± 0.3355	3	2	2.5000	2
8.	V4	− 56.9943 ± 0.6393	1	5	3.0000	3
					Consensus index	0.9265
					Trust level	0.9265

The features are extracted using Swin model and naive bayes is used as a classifer.

### Ranking of transfer function based FOX-optimization using HQ based ensemble ranking for feature selection

Eight binary FOX-optimization algorithms are compared using an ensemble ranking technique based on the half quadratic theory. The performance ranking is initially assessed using A-TOPSIS and H-TOPSIS techniques, which are suitable for ranking the alternatives obtained from probability distribution functions. The ranks obtained are further finalized by ensemble technique. The H-TOPSIS and A-TOPSIS techniques are employed to determine the ranking of the binary variants for feature selection. The calculated ranks can be found in Table 1, displays the final ranks obtained through an ensemble approach for DieT-based ultrasound features. The best mean value of the cost function obtained is -53.9172 using $V_4$, whereas the least standard deviation value of 0.3509 is obtained using the $V_1$ transfer function.

The same procedure is followed for Swin transformer based feature extraction from ultrasound images and ranks are initially evaluated using H-TOPSIS and A-TOPSIS and final ranks are obtained using HQ based ensemble techniques. The H-TOPSIS and A-TOPSIS and final ranks are provided in Table 2. With $V_1$ transfer function, the FOX optimization achieved the best cost value and least standard deviation of -67.0822 and 0.2613, respectively.

**Figure 4:**
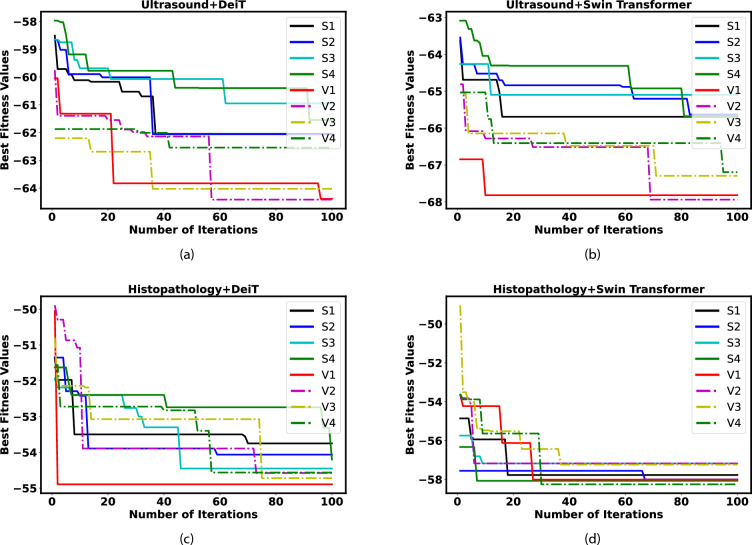
Convergence curve plots for all binary variants of FOX-algorithms using both feature extraction techniques (DeiT and Swin Transformer) for both datasets.

In the context of the histopathology dataset, the DeiT model is utilized for the purpose of features extraction. The features extracted are then subjected to evaluation with the help of H-TOPSIS and A-TOPSIS methods, resulting in the computation of initial rank vectors. These vectors serve as inputs for the final rank calculation process, incorporating a half-quadratic ensemble technique. Ultimately, the resulting final ranks are presented in Table 3. Using $V_4$ FOX optimization, the model obtained the optimal cost value of -53.9172 and the lowest standard deviation value obtained using $V_1$, which is 0.3509.

The ranks obtained from the Swin Transformer model are assessed using a similar procedure. The rankings based on H-TOPSIS, A-TOPSIS, and the final ensemble orders are displayed in Table 4. When employing $S_4$ FOX optimization, the model achieved the best cost value of -57.0820, while the smallest standard deviation value, recorded with $V_1$, is 0.3324. Figure 4 depicts the convergence curves for all proposed eight binary S and V-shaped FOX algorithms. The convergence curves are presented for both the histopathology and ultrasound datasets. For each dataset, two case studies are conducted, one using DeiT-based feature extraction and the other using Swin Transformer-based feature extraction. It is clear from the cost values that using the Swin transformer for feature extraction results in a better cost value than DeiT. The $V_1$ based FOX optimization ranked best for all the analyses, and its convergence is faster among all algorithms. In order to further improve the performance, the number of epochs increased to 1000 for $V_1$ FOX optimization, and performance is evaluated for the Swin transformer with feature selection. For the ultrasound dataset with 1000 epochs, the best values of Accuracy, F2 score, AUC-ROC, inference time, and the cardinality of feature space obtained are 94.7506%, 0.9366, 0.9848, 0.0353 second and 5. The best cost value obtained is − 75.5040. Likewise, for the histopathological dataset, the model achieved the highest values for Accuracy (89.7100%), F2 score (0.8760), AUC-ROC (0.9329), inference time (0.0514 seconds), and the feature space size, which is 12. Additionally, the optimal cost value obtained is -75.7800.

## Discussion

The outcomes presented in results section serve as a basis for evaluating the effectiveness of the proposed binary versions of FOX-optimization algorithms. This evaluation is conducted on two datasets related to thyroid cancer. The features are initially extracted using both the DeiT and Swin Transformer models. The LLE feature transformation technique transforms these extracted features into a latent space. Eight S and V-shaped binary FOX-optimization algorithms are introduced to address the feature selection aspect. A wrapper-based feature selection technique is employed, where the performance of the FOX algorithm binary variants is evaluated using Naive Bayes as a classifier. The feature selection process is treated as a multi-objective optimization problem. The performance metrics encompasses various factors such as Accuracy, F2-score, AUC-ROC score, selected features, Inference time during testing, and elapsed Time of each iteration. These algorithms are executed 30 times and mean and standard deviation values are computed and documented in Section 3 across several tables. While statistical tests are typically employed to assess the performance of optimization algorithms, they only indicate similarities between algorithms and cannot rank them. To address this limitation, the H-TOPSIS and A-TOPSIS methods are employed to calculate ranks. It is worth noting that these two methods may yield different rank values, even when provided with the same input parameters. To mitigate this issue, an ensemble ranking approach is utilized, where the rank vectors from both H-TOPSIS and A-TOPSIS are combined. This process results in final ranks, Consensus Index, and Trust Level, provided for different studies in Section 3. Upon examining the ensemble ranking tables, it becomes evident that the proposed binary FOX-optimization algorithm employing the $V_1$ shape transfer function achieves the best performance and secures the top rank for both feature extraction techniques in the final ranking process. In contrast, the binary FOX algorithm using the $S_4$ transfer function demonstrates the poorest performance for the ultrasound dataset, while the binary FOX algorithm using $S_3$ transfer function is ranked last for the histopathology dataset. The convergence curves of the proposed S and V shaped binary FOX algorithms provide additional evidence that the $V_1$ transfer function-based FOX algorithm exhibits faster convergence compared to the other variants. This suggests that the $V_1$ transfer function contributes to improved optimization performance, leading to quicker convergence towards the optimal solution. The Swin transformer based feature extraction technique provided the best cost function values with $V_1$ FOX optimization as a feature selection tool. The study performed by Chi et al.^8^ obtained 79.36% accuracy, whereas Sai et al. achieved 79.36% and 77.57% accuracies using GoogleNet and VGG16 models, respectively. In their research, Nguyen et al.^10,11^ obtained accuracies of 90.88% and 92.05% using weighted cross-entropy and ensemble CNN models. Sharma et al.^5^ achieved 82.16% accuracy with transformer and mixer-based ensemble models on the ultrasound dataset. Most of the studies only tried to improve the performances irrespective of time complexity. In our research, the inference time is also studied along with different performance metrics. We try to reduce the feature space size to reduce overfitting as a multi-objective optimization problem. The proposed model outperformed current leading methods for the ultrasound dataset and achieved 94.75% accuracy. Similarly, the proposed technique achieved results comparable to existing techniques for the histopathological dataset. In the Do et al.^16^ research, they obtained an accuracy of 85.73% using the Inception V-3 model whereas Bohland et al.^15^ obtained 89.10% using the deep CNN model. The proposed model achieved 89.71% accuracy, comparable with the existing research for histopathological. The performance of the optimization algorithms is evaluated on only two datasets, even with different modalities. It is due to the scarcity of medical image datasets in the thyroid domain due to patient participation and the burden on healthcare.

## Conclusion

The binary variant of the FOX algorithm is derived by converting the continuous version using either S-shaped or V-shaped transfer functions. These proposed techniques offer a valuable solution for feature selection in machine learning, allowing the evaluation of different algorithms’ search capabilities. The feature selection problem is formulated as a multiobjective problem, incorporating dimensionality reduction, classification accuracy, F2-score, AUC-ROC, and Inference Time. The binary variants are evaluated on two distinct datasets: one related to thyroid ultrasound cancer images and the other to histopathology cancer images. An ensemble MCDM ranking technique is employed to rank the binary variants, specifically for comparison in this study. It’s important to note that the proposed methodology can be applied to various healthcare image datasets, facilitating performance evaluation in different scenarios. Additionally, alternative MCDM techniques can be employed for ranking purposes, providing further flexibility in the evaluation process. Furthermore, it’s worth noting that the proposed framework holds potential for application in quantum machine learning and federated learning, although these areas are not within the scope of this work. They serve as interesting directions for future research and development, highlighting the versatility and adaptability of the proposed framework. The trust level and consensus index have equal values because we only considered two initial ranking techniques. However, other MCDM techniques are available, such as VIKOR and PROMETHEE, that can be employed to rank the performances of the algorithms.

## Data Availability

The histopathology Tharun and Thompson dataset is provided on request by Dr. Lars Tharun and Dr. Lester Thompson. The source of dataset is https://pubmed.ncbi.nlm.nih.gov/29508145 with PubMed ID: 29508145.
